# ¿Compulsion or perseveration? A case report

**DOI:** 10.1192/j.eurpsy.2022.1661

**Published:** 2022-09-01

**Authors:** A.M. Ruiz Moliner, A. Pérez Romero, A. Carranza Pérez-Tinao, S. González Garrido

**Affiliations:** Hospital Universitario Virgen Macarena, Ugc Salud Mental, Sevilla, Spain

**Keywords:** Obsessive rituals, frontotemporal dementia, obsessive-compulsive disorder, perseveration

## Abstract

**Introduction:**

54-year-old female patient who came to hospital due to psychopathological decompensation of her Obsessive-Compulsive Disorder (OCD), after 35 years under follow-up. Parkinson´s disease. Psychopharmacological treatment: sertraline 100 mg (1-0-0); lorazepam 2 mg (1-1-1); Levodopa/carbidopa 100/25 mg (1-1-1). Distressed at first examination. She described increase in rituals, important intake restriction, weight impact and difficulties in home management with functional repercussions. Psychopathological exploration: conscious, oriented, and approachable. Circumstantial speech with no obsessive ideas. Increased frequency of repetitive behaviours led to a functional deterioration, becoming dependent for activities of daily living. Elevated anxiety. No major mood disorder. No psychotic symptoms. Bradykinesia. Hypophagia without anorexia. Admission is carried out. Good evolution: improvement in motor symptoms and intake restoration. No changes in repetitive behaviours.

**Objectives:**

To discuss the differential diagnosis between OCD and Frontotemporal Dementia.

**Methods:**

Repetitive behaviours were initially understood as rituals typical of OCD. However, the absence of both a fixed pattern of behaviour and a structured obsessive ideation, made us consider the possibility of frontal perseveration behaviours. For this reason, a neuropsychological evaluation and a functional neuroimaging test were performed: Test Mo-CA: 9/30 with striking failures in executive functions. SPECT: mild uptake defect in the left frontotemporal region.

**Results:**

Finally, in view of the impairment in executive functions and the frontal defects in neuroimaging, we change the initial diagnosis of OCD towards a Neurocognitive Disorder of probable frontotemporal origin.

**Conclusions:**

The presented case evidenced the importance of differentiating obsessive compulsions from frontal perseverance to guide the differential diagnosis, given the implications for therapeutic management and prognosis.

**Disclosure:**

No significant relationships.

